# An investigation of mental imagery in bipolar disorder: Exploring “the mind's eye”

**DOI:** 10.1111/bdi.12453

**Published:** 2016-12-20

**Authors:** Martina Di Simplicio, Fritz Renner, Simon E Blackwell, Heather Mitchell, Hannah J Stratford, Peter Watson, Nick Myers, Anna C Nobre, Alex Lau‐Zhu, Emily A Holmes

**Affiliations:** ^1^ Medical Research Council Cognition and Brain Sciences UnitCambridgeUK; ^2^ Highfield Inpatient Adolescent UnitWarneford HospitalOxfordUK; ^3^ Oxford Centre for Human Brain Activity (OHBA)University of OxfordOxfordUK; ^4^ Department of Experimental PsychologyUniversity of OxfordOxfordUK; ^5^ Department of Clinical NeuroscienceKarolinska InstituteStockholmSweden; ^6^ Mental Health Research and Treatment CenterRuhr‐Universität BochumBochumGermany

**Keywords:** affective lability, anxiety, bipolar disorder, cognitive functioning, depression, mental imagery

## Abstract

**Objectives:**

Mental imagery abnormalities occur across psychopathologies and are hypothesized to drive emotional difficulties in bipolar disorder (BD). A comprehensive assessment of mental imagery in BD is lacking. We aimed to test whether (i) mental imagery abnormalities (abnormalities in cognitive stages and subjective domains) occur in BD relative to non‐clinical controls; and (ii) to determine the specificity of any abnormalities in BD relative to depression and anxiety disorders.

**Methods:**

Participants included 54 subjects in the BD group (depressed/euthymic; n=27 in each subgroup), subjects with unipolar depression (n=26), subjects with anxiety disorders (n=25), and non‐clinical controls (n=27) matched for age, gender, ethnicity, education, and premorbid IQ. Experimental tasks assessed cognitive (non‐emotional) measures of mental imagery (*cognitive stages*). Questionnaires, experimental tasks, and a phenomenological interview assessed subjective domains including spontaneous imagery use, interpretation bias, and emotional mental imagery.

**Results:**

(i) Compared to non‐clinical controls, the BD combined group reported a greater impact of intrusive prospective imagery in daily life, more vivid and “real” negative images (prospective imagery task), and higher self‐involvement (picture‐word task). The BD combined group showed no clear abnormalities in cognitive stages of mental imagery. (ii) When depressed individuals with BD were compared to the depressed or anxious clinical control groups, no significant differences remained—across all groups, imagery differences were associated with affective lability and anxiety.

**Conclusions:**

Compared to non‐clinical controls, BD is characterized by abnormalities in aspects of emotional mental imagery within the context of otherwise normal cognitive aspects. When matched for depression and anxiety, these abnormalities are not specific to BD—rather, imagery may reflect a transdiagnostic marker of emotional psychopathology.

## 1 Introduction

Mental imagery comprises the experience of seeing in the “mind's eye,” now regarded as “a weak form of perception”.^1^ No wonder that negative mental images generate strong emotions, indeed stronger than does thinking in verbal language.^2^

Bipolar disorder (BD) is characterized by periods of heightened emotion (depression and mania),^3^ both during acute episodes and inter‐episodically.^4^, ^5^ We have suggested that mental imagery may act as an “emotional amplifier”—fueling mood deterioration, mood elevation, and anxiety symptoms typical in BD.^6^ Initial data suggested that patients with BD present with heightened emotional mental imagery compared to non‐clinical controls,^7^ in particular higher trait imagery use and heightened impact of intrusive mental imagery of future events (prospective imagery). Furthermore, those patients with BD with greatest mood instability reported a greater impact of prospective imagery.^7^ Compared to unipolar patients with equivalent levels of depressed mood, patients with BD reported more compelling and preoccupying prospective suicidal images.^8^ This is of interest given that BD has the highest suicide rate of all psychiatric disorders.^9^ Patients with BD also reported more frequent “flashforwards” to future events at times of positive mood than did people with unipolar depression, and rated these “flashforwards” as more vivid, exciting, and pleasurable.^10^

However, a more comprehensive assessment of mental imagery function in BD is lacking. Pearson et al.^11^ argued for complementing clinical measurements of imagery with more traditional cognitive (non‐emotional) measures, and for assessment using both objective cognitive stage measures (non‐emotional) and subjective domain measures (emotional). The cognitive stages are based on a computational theory proposed by Kosslyn et al.^12^ concerning four main stages of mental imagery: generation, maintenance, inspection and manipulation. Previous studies have investigated only selected stages of imagery‐related processing, with evidence of deficits in cognitive tasks of imagery generation and manipulation in depressed individuals^13^ and imagery generation in anxious individuals.^14^ The subjective domains relate to spontaneous imagery use,^15^ the presence of imagery‐related interpretation biases and emotional mental imagery^16^, ^17^, ^18^ and the phenomenological characteristics of mental imagery in different affective states.^16^

“Rediscovering” mental imagery in clinical practice can improve assessment.^17^ There is also emerging evidence of imagery as a valid target to reduce mood instability in BD.^18^ Therefore, a comprehensive evaluation of cognitive stages and subjective domains of mental imagery in BD could further inform our understanding of BD psychopathology and treatment development, by identifying problematic aspects of mental imagery in BD and refining treatment targets.

The current study aimed to investigate: (i) whether individuals with BD have mental imagery abnormalities compared to non‐clinical controls and (ii) whether mental imagery abnormalities (when present) are specific to individuals with BD compared to clinical controls with depression and anxiety. To address these questions, we compared (i) patients with BD and non‐clinical controls; (ii‐a) patients with BD and patients with unipolar depression with equivalent levels of depressive symptoms; and (ii‐b) patients with BD and patients with anxiety disorders with equivalent levels of anxiety symptoms. We also explored whether clinical variables such as depressive and anxious symptomatology, bipolar phenotype traits, affective lability and general functioning levels predicted scores on mental imagery measures in the whole sample combined. A range of tests were used encompassing both cognitive stages of mental imagery and assessment of subjective and emotional domains.

## 2 Materials and Methods

### 2.1 Participants

Participants completed pre‐screening questions via email or phone to assess potential eligibility, based on which 175 were invited to attend a screening session. At the beginning of the session, all participants provided written informed consent (ethical approval reference: REC South Central 11/SC/0182) and the Structured Clinical Interview for DSM‐IV (SCID) Axis I Disorders^19^ was administered to establish diagnosis. The testing battery included questionnaires, experimental tasks and a phenomenological interview (average duration 4 h). If participants were unable to complete testing over one session, a second session was scheduled where mood state was reassessed.

The screening session was used to determine whether participants met the following DSM‐IV diagnostic criteria: *BD group:* DSM‐IV diagnosis of bipolar I disorder, bipolar II disorder, or bipolar disorder not otherwise specified, not current (hypo)manic episode; *unipolar depression group:* DSM‐IV diagnosis of major depressive episode (MDE); *anxiety disorder group:* DSM‐IV diagnosis of anxiety disorder in the absence of a present or past history of BD and of a current primary MDE; *non‐clinical control group:* no past or present Axis I disorder based on DSM‐IV diagnosis. Exclusion criteria for all participants were active suicidal risk, psychotic symptoms, current substance abuse, all assessed during the SCID, and severe neurological impairment reported during the screening session. Of the 175 participants who attended the screening session, 24 were excluded based on these criteria.

Allocation to one of the experimental groups was confirmed by a clinician (in the case of queries about the SCID), corroborated by scores of current affective state (i.e., score of ≥8 on the Hamilton Rating Scale for Depression [HAM‐D]^20^ to indicate current depression; score of <8 on the HAM‐D to indicate euthymia; no change in affective state between testing sessions). Participants with a diagnosis of BD were allocated to the “BD depressed” or to the “BD euthymic” group on the basis of the SCID (i.e., current MDE or no current MDE) and HAM‐D scores. A further 18 participants were excluded from analysis, based on a HAM‐D score inconsistent with the SCID interview (n=6), a change in mood state across testing sessions (n=1), or a further check by the clinician of the SCID interview/additional information indicating that the individual was not eligible (e.g., current substance dependence, not meeting SCID criteria for experimental group, or current (hypo)mania; n=11). Two participants did not complete the testing sessions.

The final sample analyzed consisted of 131 participants, comprising individuals with BD (depressed [n=27] and euthymic [n=27]), unipolar depression (n=26), or anxiety disorders (n=25), and 26 non‐clinical controls, aged 18–65 years.

### 2.2 Assessments

#### 2.2.1 Clinical characteristics

Clinical characteristics were assessed using the SCID for DSM‐IV Axis I disorders, including main diagnosis and lifetime and current comorbid disorders, as above. Current medication was recorded. Depressive, (hypo)manic, and anxiety symptoms were assessed using the HAM‐D, the Young Mania Rating Scale,^21^ the Altman Self‐Rating Mania scale,^22^ the Quick Inventory of Depressive Symptomology (QIDS),^23^ and the Beck Anxiety Inventory (BAI).^24^ The Mood Disorder Questionnaire (MDQ)^25^ was administered to assess hypomanic experience. The Affective Lability Scale (ALS)^26^ was used to measure changeable affect and the Functional Assessment Staging Test^27^ to assess functional impairment in areas including occupational functioning, cognitive functioning, and interpersonal relationships.

#### 2.2.2 General cognitive function

The National Adult Reading Test^28^ was used as an assessment of premorbid IQ. Verbal fluency (as a measure of general executive function) and verbal working memory function were assessed using the Verbal Fluency Test with the letters F, A, S^29^ and Forward and Backward Digit Span Task,^30^ respectively.

#### 2.2.3 Subjective domain of mental imagery

##### Spontaneous imagery use

The spontaneous use of mental imagery in everyday life was assessed via the Spontaneous Use of Imagery Scale (SUIS)^15^ and two Visual Analogue Scales (VASs).^7^ The SUIS is a 12‐item self‐report scale measuring the use of non‐emotional mental imagery in daily life (e.g., *If I am looking for new furniture in a store, I always visualize what the furniture would look like in particular places in my home*.). Each item is rated on a five‐point scale, with total scores ranging from 12 to 60. Higher scores indicate more use of mental imagery in daily life. The SUIS has an internal consistency of α=0.98 and good convergent validity.^15^ Two VASs were used to assess the extent to which participants had been thinking in verbal thoughts or in mental images over the past week on a 1 (*not at all*) to 9 (*all the time*) scale.

##### Imagery interpretation bias

The Ambiguous Scenarios Test (AST‐D)^31^ and the Homograph Interpretation Task (HIT)^32^ were used to measure imagery interpretation bias. The AST‐D comprises 24 ambiguous scenarios, which participants were asked to imagine happening to them personally (e.g., *1=You go to a wedding where you know very few other guests. After the party, you reflect on how the other guests behaved*.), and then rate each image's pleasantness from 1 (*extremely unpleasant*) to 9 (*extremely pleasant*) and vividness from 1 (*not at all vivid*) to 7 (*extremely* vivid). The AST‐D has good internal consistency (α=0.82).^31^ In the HIT, participants are presented with a word and then asked to generate a mental image. The words were eight threatening/non‐threatening homographs, for example, “mug” could cue either a benign (e.g., imagining oneself drinking out of a mug) or negative (e.g., imagining being attacked/mugged) mental image. Participants provided a short written description of each image and then rated their pleasantness (1–9 scale) and vividness (1–7 scale). Average vividness and pleasantness scores were computed for benign, negative and ambiguous mental images.

##### Emotional mental imagery

Emotional mental imagery was assessed using a Picture Word Cue (PW) task,^33^ the Impact of Future Events Scale (IFES),^34^ and the Prospective Imagery Task (PIT).^32^, ^35^ The Mental Imagery Interview (MII) (modified from Ref. 8) was conducted to gain qualitative descriptions of the phenomenology of images and verbal thought at times of different acute affective states (low, elated, and anxious affect).

The PW task is a computer‐based task examining self‐reported spontaneous use of imagery in response to emotional information and emotional context. Participants were presented with 20 ambiguous/neutral pictures with negative word captions and instructed to “combine the picture with the word” (e.g., picture of students sitting an exam and caption word “fail”). They then rated from 1 (*not at all*) to 9 (*extremely*) how much they found themselves thinking in mental images, or in verbal thoughts, and how emotional they found the picture−word combination. Average tendency to use images and verbal thoughts and average emotionality of the picture−word combinations were computed.

On the IFES, participants were asked to identify three future events they had thought about/imagined over the past 7 days and state whether each was positive or negative. Participants then responded to 24 statements about prospective imagery in relation to the past week, on a scale from 0 (*not at all*) to 4 (*extremely*). The IFES has acceptable test−retest reliability (0.73) and a good internal consistency (α=0.87).^34^

The PIT comprises 10 positive and 10 negative hypothetical future scenarios. Participants were asked to generate an image of each and rate each image on a five‐point Likert scale for vividness, likelihood of the event happening to them in the near future, and how much they feel as though they are experiencing the event whilst imagining it, with higher ratings indicating more vivid and “real” prospective imagery. All subscales of the PIT have demonstrated good internal consistency (0.83<α<0.90).^36^

The MII is a semi‐structured interview, which assesses content and characteristics of mental images and verbal thoughts experienced when the participant has been most anxious, most low and most high in mood. Participants are first asked to describe their most significant mental image anchored to each affect state and rate characteristics of the image such as valence, general emotionality of the image and intensity of one specific associated emotion per each affect state (i.e., threatening, demotivating and exciting). They are then asked to rate overall characteristics of mental imagery and verbal thoughts for each affect state (anxious, low, and high) such as frequency, realness, and compellingness. All ratings use nine‐point Likert scales, with higher ratings indicating more frequent, real (etc.) imagery or thoughts.

#### 2.2.4 Cognitive (non‐emotional) stages of mental imagery

The following tasks were administered to assess the four cognitive stages of mental imagery.^11^

##### Imagery generation

The *Image Generation Task* (IGT)^37^ measures the ability to generate a mental image based on previously encountered perceptual information. Participants were asked to memorize the shape of four block capital letters presented in a 4 × 5 grid: “U” and “H”, classified as simple (three or fewer segments), and “S” and “J,” classified as complex (four or more segments). Participants were then presented with a blank grid with a lowercase letter underneath, indicating which letter the participant should imagine. An “X” was presented in one of the grid squares and participants were asked to respond “True” if the “X” would cover the imagined block letter if it were present in the grid or otherwise “False.” Accuracy and reaction time were recorded. Socially anxious participants have previously shown image generation deficits on this task.^14^

##### Imagery maintenance

The ability to maintain mental images in mind was assessed using two visual working memory tasks. The Short Term Memory (STM) task (adapted from Ref. 38) measures visual working memory capacity as the number of items that can be maintained in a mental representation as well as the quality of representations.^39^ Participants were presented with arrays consisting of four arrows at different orientations. A test arrow was then presented at one of the previous four locations in a random orientation, and participants were instructed to respond by moving the mouse up or down to rotate the test arrow clockwise or anticlockwise until it matched their memory for the remembered arrow and then to confirm their response. Visual feedback was provided immediately afterwards. The angular deviation between the participants’ selected orientation and the original orientation of the arrow provided a measure of the error in the participants’ memory for the scene. The distribution of angular errors across trials was used to compute (using a well‐established modeling technique^39^) two complementary accuracy measures: recall rate and memory precision. Recall rate reflects the proportion of trials on which participants have at least some information in mind about the remembered stimulus, whereas memory precision reflects how clear that information is.

The *Visual Patterns Test* (VPT)^40^ measures visual short‐term memory and memory for positional sequences. Participants were presented with a sequence of increasingly complex checkerboard patterns, starting with a 2 × 2 matrix (with two cells filled in) and progressing to the largest 5 × 6 matrix (with 15 filled in cells). Each pattern was shown to the participant for 3 s and then hidden, at which point participants were asked to reproduce the pattern by marking squares in an empty grid of the same size. Accuracy scores were calculated using the maximum difficulty level reached for which two patterns were correctly reproduced.

##### Imagery inspection

The Letter Corner Classification (LCC) task^41^ measures image inspection ability, involving interpretation of an object‐based spatial characteristic of the image. Participants were first presented with four block capital letters (F, N, Z, and G), marked with an asterisk in the bottom left corner and an arrow travelling clockwise around the letter. Participants were instructed to memorize the shape of each letter and reproduce it on a blank piece of paper, starting at the point marked by the asterisk and following the direction of the arrow. Participants then categorized the corner of the letters. For each letter, first, for “top and bottom points”, participants were asked to go around the shape, starting at the point marked by the asterisk, indicating “yes” if the corner was at the extreme top or bottom of the shape or otherwise “no”. The letters were then removed and participants instructed to imagine each letter and categorize the corners. The letters were then presented again and the same procedure followed for “outside points”, which required a “yes” response for corners on the extreme left and right of the figure. Accuracy and time taken for each letter in both conditions were recorded.

##### Imagery manipulation

Two tasks measuring the ability to manipulate mental images were administered. A computerized version of the classic Mental Rotation Task (MRT)^42^ measured participants’ ability to transform mental images. Participants were shown pairs of three‐dimensional line drawings and instructed to decide whether the two drawings were the same or different by using a mental rotation strategy. Following a practice trial, the task included trials with three difficulty levels based on whether the angular disparity between the two shapes was 50, 100, or 150. Accuracy and reaction time were recorded. Two measures were computed based on:^13^, ^43^ the intercept index, representing the sensory/motor component of the response in the task, and the slope index, representing the spatial ability component of the task (i.e., the rotation speed relative to the angular rotation difficulty). A version of the MRT using letters of the alphabet^44^ has shown mental rotation deficits in patients with unipolar depression.

The Creative Mental Synthesis (CMS) task^45^ assesses participants’ ability to mentally construct a recognizable figure from three alpha‐numeric or geometric shapes (e.g., rectangle, capital L, and horizontal line). Participants were shown two sets of example mental constructions and then completed two trials. On each trial three parts were named, after which participants were given 2 min to close their eyes and mentally combine the stimuli into a recognizable figure. They were then asked to draw and label their final figure. Three judges independently rated each figure on the following parameters: recognizability (of zero, one, or two of the two trials), correspondence (between the name of the pattern and the drawing on a 1–5 scale), creativity (yes/no, for patterns rated at least 4 for correspondence), wrong patterns (yes/no) and absence of pattern (yes/no).

#### 2.2.5 Statistical analysis

First, we tested (i) if participants with BD had imagery abnormalities by comparing the BD group (euthymic and depressed combined) to non‐clinical control participants on measures of cognitive and subjective domains of mental imagery. To test for between‐group differences on these aspects of imagery abnormalities, 55 statistical tests were performed. A BD group combining euthymic and depressed individuals was used to test replication of previous data.^7^ Moreover, as euthymic individuals with BD present with depression levels greater than those of non‐clinical controls (albeit subclinical), we chose to first assess the presence of imagery abnormalities regardless of affect state. Next, we sought to determine the specificity of any group differences by comparing the scores of (ii‐a) currently depressed participants with BD to those of currently depressed participants with unipolar depression (this also allows controlling for the impact of depressed mood on mental imagery abnormalities), and (ii‐b) currently depressed participants with BD with concurrent anxiety symptoms to those of participants with anxiety disorders (this also allows controlling for the impact of anxiety on mental imagery abnormalities; the two groups were also matched on levels of depression). To limit the number of tests, comparisons of BD depressed to clinical control groups were limited to (i) those variables that showed significant group differences in the initial comparison (BD group combined versus non‐clinical controls) and (ii) those comparisons that had yielded significant differences in previous studies.^7^, ^8^ To test for differences between depressed participants with BD and unipolar depressed participants and to test for differences between depressed participants with BD with concurrent anxiety symptoms and participants with anxiety disorders, 38 statistical tests were performed.

Pairwise differences between variables in the different groups as outlined in our aims were analyzed using unpaired *t* tests if the residuals obtained using these *t* tests achieved normality with *P*‐values above .05 using both the Kolmogorov–Smirnov and Shapiro–Wilk tests. Where the group variances were found to differ using Levene's test, Satterthwaite's correction was applied to the degrees of freedom of the *t* test. Where the residuals of a pairwise comparison on an untransformed response did not achieve normality, log, square root and reciprocal transformations were applied and normality of the residuals reassessed. Where transformations failed to achieve normal residuals, Mann–Whitney *U* tests were used to analyze group differences. For the CMS task, Fisher's exact test was used to identify group differences in the number of CMS trials that were judged as recognizable, creative, having good correspondence, having a correct pattern and having a present pattern.

To explore the specificity of differences in mental imagery between diagnostic groups further, we computed correlations between the mental imagery variables that showed significant group differences in the initial comparison (BD group combined versus non‐clinical controls) and clinical variables for depression (QIDS and HAM‐D), anxiety (BAI), hypomanic experiences (MDQ), mood instability (ALS), and overall functioning (FAST). Pearson's correlations were used, or Kendall's tau, where inspection of scatterplots did not suggest a linear relationship (96 correlations computed). The unique contribution of these clinical variables in predicting scores on the mental imagery measures across groups was explored by conducting a series of multiple regression analyses with each of the mental imagery measures as the dependent variable (16 regression models) and with all clinical variables entered as predictor variables simultaneously. Non‐significant predictors were then removed from the model stepwise until only significant predictors remained. Mania measures were not included in this analysis as all participants presented with levels of manic symptoms below clinical significance (see the *Limitations* section).

In all analyses, *P*‐values <.05 were considered statistically significant and no corrections for multiple testing were applied. Normality checks of model residuals allowed any undue influence of outliers to be reduced without losing information by removing them.

## 3 Results

### 3.1 Participants

Demographic and clinical characteristics of all groups are presented in Table 1. There were no between‐groups differences in age, gender, ethnicity, level of education, or premorbid IQ.

**Table 1:** Demographic and clinical characteristics of participants

	BD (euthymic) (n=27)	BD (depressed) (n=27)	Unipolar depression (n=26)	Anxiety disorder (n=25)	Non‐clinical controls (n=26)
**Demographic characteristics**					
Age, years, mean (SD)	40.41 (12.78)	40.44 (12.56)	44.31 (14.82)	37.60 (15.43)	41.50 (13.00)
Educational level, years, mean (SD)	17.11 (2.64)	17.07 (4.07)	17.27 (3.97)	16.52 (3.00)	17.46 (2.28)
Gender, female, n (%)	17 (63.0)	17 (63.0)	18 (69.2)	19 (76.0)	16 (61.5)
Estimated premorbid IQ, mean (SD)	114.85 (7.96)	113.04 (9.19)	112.20 (11.50)	111.42 (9.44)	112.88 (10.75)
*Ethnicity*					
White	26	25	17	21	24
Mixed	0	1	3	2	1
Asian or British Asian	0	0	3	1	1
Chinese	1	1	3	0	0
**Clinical characteristics**					
Bipolar I disorder, n (%)	17 (63.0)	14 (51.9)	0	0	0
Bipolar II disorder, n (%)	10 (37.0)	11 (40.7)	0	0	0
BP‐NOS, n (%)	0 (0.0)	2 (7.4)	0	0	0
No. of depressive episodes, mean (SD)	19.05 (23.67)	22.76 (30.81)	4.81 (9.34)	14.15 (29.97)	0
Current depression, n (%)	0	27 (100.0)	26 (100.0)	8 (32.0)	0
Current anxiety disorder, n (%)^a^	6 (22.2)	16 (59.3)	11 (42.3)	25 (100.0)	0
*Medications, n*					
Antidepressants	8	8	7	9	0
Anxiolytics	2	3	0	2	0
Mood stabilizers	19	13	0	0	0
Antipsychotics	14	10	1	0	0
History of Axis I disorders					
Previous depression, n	27	25	22	17	0
Previous anxiety, n	9	11	7	11	0
Previous other, n	13	12	5	1	0
Age at illness onset, years, mean (SD)	21.30 (10.01)	16.81 (8.38)	26.75 (13. 10)	19.48 (13.18)	n/a
Illness duration, years, mean (SD)	18.67 (11.93)	23.77 (15.44)	15.88 (16.17)	17.38 (15.46)	n/a
**Current clinical symptoms, mean (SD)**					
QIDS score	4.37 (2.82)	13.22 (3.93)	15.50 (4.47)	11.32 (5.71)	2.04 (2.01)
HAM‐D score	3.19 (2.18)	14.93 (4.59)	15.23 (4.99)	11.40 (7.05)	1.31 (1.49)
BAI score	3.62 (3.74)	14.41 (9.01)	16.38 (10.33)	17.96 (9.06)	2.00 (2.87)
ASRM score	2.96 (2.79)	1.59 (2.58)	1.28 (1.81)	1.88 (1.83)	0.88 (1.11)
YMRS score	2.65 (2.38)	2.81 (3.71)	1.88 (1.93)	2.56 (1.89)	0.42 (0.86)
FAST score	7.67 (6.74)	26.44 (11.92)	31.12 (14.53)	23.56 (16.29)	4.69 (9.44)
ALS score	62.48 (33.56)	82.58 (24.54)	67.69 (26.40)	70.80 (32.59)	22.23 (19.62)
MDQ score	14.41 (2.00)	13.07 (2.89)	7.88 (4.93)	6.04 (4.11)	2.46 (2.89)

ALS, Affective Lability Scale; ASRM, Altman Self‐Rating Mania scale; BAI, Beck Anxiety Inventory; BD, bipolar disorder; BD‐NOS, bipolar disorder not otherwise specified; FAST, Functional Assessment Staging Test; QIDS, Quick Inventory of Depressive Symptomology; HAM‐D, Hamilton Depression Rating Scale for Depression; MDQ, Mood Disorder Questionnaire; SD, standard deviation; YMRS, Young Mania Rating Scale.

a Current anxiety disorder types were: social anxiety (n=12), obsessive compulsive disorder (n=9), posttraumatic stress disorder (n=11), generalized anxiety disorder (n=20), specific phobia (n=10), panic disorder (n=12), and agoraphobia (n=2). Please note that some participants presented with multiple anxiety disorders.

### 3.2 Assessments

#### 3.2.1 General cognitive function

Participants with BD (combined group) had a lower total score on the verbal fluency task (mean [M]=41.30, standard deviation [SD]=10.56) compared to non‐clinical controls (M=48.85, SD=15.29); *t*(78)=2.58, *P*=.012, *d*=0.62. The two groups did not differ in their performance on the digit span task: digit span forward *P*=.94; digit span backwards *P*=.72. There were no differences in any of the general cognitive functioning measures between the BD depressed group and the unipolar depression group (verbal fluency task *P*=.66; digit span forward *P*=.39; digit span backward *P*=.36) or between the BD depressed group and the anxiety disorders group (verbal fluency task *P*=.07; digit span forward *P*=.83; digit span backward *P*=.95).

### 3.3 Do individuals with BD show mental imagery abnormalities compared to non‐clinical controls?

Scores on assessments of cognitive (non‐emotional) stages and subjective domains of mental imagery of participants with BD and non‐clinical controls, and results of between‐group comparisons are summarized in Tables 2, 3, 4 (all data referring to the BD combined group).

**Table 2:** Mean differences between participants with bipolar disorder and non‐clinical control participants in measures relating to the cognitive (non‐emotional) stages of mental imagery

	Bipolar disorder	Non‐clinical controls					
	Mean (SD) (n=54)	Mean (SD) (n=24)	*t*	*Z*	df	*P*‐value	*d*
**Imagery generation**							
*Imagery Generation Task (IGT)*							
IGT RT Simple Letter	2026.48 (842.65)	1744.22 (662.54)	—	1.04	—	0.30	0.36
IGT RT Complex Letter	2175.07 (914.24)	1843.46 (568.48)	1.64	—	76	0.11	0.40
IGT RT Early	1964.54 (761.72)	1767.53 (575.63)	1.13	—	76	0.26	0.28
IGT RT Late	2250.91 (1001.15)	1896.33 (602.24)	1.61	—	76	0.11	0.39
IGT Percentage Errors	4.40 (11.04)	3.39 (5.21)	—	0.22	—	0.83	0.10
**Imagery maintenance**							
*Short‐term Memory Task (STM)*							
STM Memory Precision	3.12 (3.93)	2.65 (0.83)	—	0.41	—	0.68	0.14
**STM Recall Rate**	0.65 (0.21)	0.53 (0.19)	2.15	—	67	0.04*	0.58
*Visual Pattern Task (VPT)*							
VPT Accuracy	9.25 (1.67)	9.43 (1.76)	0.45	—	75	0.66	0.11
**Imagery Inspection**							
*Letter Corner Classification Task (LCC)*							
LCC Accuracy	5.30 (1.88)	5.11 (1.91)	—	0.50	—	0.62	0.10
LCC Time	14.50 (6.64)	13.02 (5.13)	—	0.99	—	0.32	0.25
**Imagery manipulation**							
*Mental Rotation Task (MRT)*							
MRT RT Easy	3080.64 (722.74)	3015.35 (588.61)	0.39	—	73	0.70	0.10
MRT RT Medium	3389.18 (697.81)	3265.71 (588.76)	0.76	—	73	0.45	0.18
MRT RT Difficult	3510.54 (642.93)	3261.41 (602.33)	1.62	—	73	0.11	0.40
MRT slope	214.81 (175.40)	133.04 (166.17)	1.94	—	73	0.06	0.47
MRT intercept	2880.19 (810.89)	2933.09 (652.66)	0.28	—	73	0.78	0.07
**MRT Percentage Errors**	28.22 (14.54)	20.62 (10.85)	2.31	—	73	0.02*	0.57

RT, reaction time; SD, standard deviation. *P<0.05.

**Table 3:** Mean differences between participants with bipolar disorder and non‐clinical control participants on measures relating to subjective domains of mental imagery

	Bipolar disorder	Non‐clinical controls					
	Mean (SD)	Mean (SD)	*t*	*Z*	df	*P*‐value	*d*
**Spontaneous imagery use**							
*Spontaneous Use of Imagery Scale (SUIS)*							
SUIS mean score	3.36 (0.81)	3.04 (0.65)	1.75	—	78	0.08	0.42
Visual Analogue Scales (VASs)							
VAS Verbal	5.38 (2.21)	5.73 (1.80)	—	0.62	—	0.53	0.17
VAS Mental Imagery	5.38 (2.11)	5.31 (1.49)	—	0.39	—	0.70	0.04
**Imagery interpretation bias**							
*Ambiguous Scenarios Test (AST‐D)*							
AST‐D Pleasantness	4.83 (1.11)	5.17 (1.23)	1.23	—	78	0.22	0.30
AST‐D Vividness	4.45 (1.38)	4.60 (1.37)	0.48	—	78	0.63	0.11
*Homograph Interpretation Task (HIT)*							
HIT no. of Positive Homographs	4.91 (1.69)	5.62 (1.55)	—	1.91	—	0.06	0.43
HIT no. of Negative Homographs	2.89 (1.69)	2.27 (1.51)	—	1.60	—	0.11	0.38
HIT Positive Vividness	5.09 (1.30)	5.19 (1.16)	—	0.26	—	0.79	0.08
HIT Negative Vividness	4.50 (1.94)	4.32 (2.04)	—	0.44	—	0.66	0.09
**Emotional mental imagery**							
*Picture Word Task (PW)*							
Mental Imagery	6.21 (1.68)	5.92 (1.81)	0.70	—	77	0.49	0.17
Verbal	3.80 (1.97)	4.29 (2.34)	0.96	—	77	0.34	0.23
Memory	4.20 (1.45)	3.53 (1.47)	1.91	—	77	0.06	0.46
Emotion	4.77 (1.82)	4.53 (1.74)	0.55	—	77	0.59	0.13
Self‐involvement	4.44 (1.69)	3.58 (1.75)	2.10	—	77	0.04*	0.50
*Impact of Future Events Scale (IFES)*							
IFES Total Score	29.87 (15.82)	17.42 (9.31)	4.36	—	73.83	<0.001*	0.89
*Prospective Imagery Task (PIT)*							
PIT Negative Vividness	3.07 (0.92)	2.53 (0.86)	2.55	—	78	0.013	0.60
PIT Negative Likelihood	2.58 (0.67)	2.32 (0.66)	1.66	—	78	0.10	0.39
PIT Negative Experiencing	2.66 (0.91)	2.18 (0.90)	2.18	—	78	0.03*	0.53
PIT Positive Vividness	3.08 (0.91)	3.33 (0.72)	1.33	—	61.49	0.19	0.29
PIT Positive Likelihood	2.89 (0.91)	3.43 (0.69)	2.93	—	63.29	0.005*	0.64
PIT Positive Experiencing	2.59 (0.97)	2.87 (0.85)	1.23	—	78	0.22	0.30

SD, standard deviation. *P<0.50.

**Table 4:** Mean differences between participants with bipolar disorder and non‐clinical control participants in measures of imagery and thought characteristics in daily life at times of acute affect (anxiety and low and high mood) obtained from the Mental Imagery Interview

	Bipolar disorder	Non‐clinical controls			
	Mean (SD)	Mean (SD)	*Z*	*P*‐value	*d*
**Low mood**					
*Imagery in daily life*					
Frequency	5.50 (2.36)	4.81 (2.38)	1.08	0.28	0.29
Real	6.67 (2.48)	6.32 (2.46)	0.75	0.46	0.14
Compelling	6.19 (2.67)	6.04 (2.44)	0.47	0.64	0.06
*Verbal thoughts in daily life*					
Frequency	5.59 (2.29)	5.68 (2.25)	0.18	0.86	0.04
Real	6.76 (2.32)	6.92 (2.13)	0.11	0.92	0.07
Compelling	5.98 (2.54)	6.38 (1.79)	0.30	0.76	0.17
**Anxious affect**					
*Imagery in daily life*					
Frequency	5.78 (2.52)	4.48 (2.58)	2.08	0.04*	0.51
Real	7.08 (2.20)	5.96 (2.35)	2.41	0.02*	0.50
Compelling	6.42 (2.41)	5.72 (2.49)	1.38	0.17	0.29
*Verbal thoughts in daily life*					
Frequency	5.54 (2.45)	5.19 (2.12)	0.66	0.51	0.15
Real	6.92 (2.18)	6.38 (2.26)	1.21	0.23	0.24
Compelling	6.28 (2.57)	5.96 (2.32)	0.88	0.38	0.13
**High mood**					
*Imagery in daily life*					
Frequency	6.37 (2.30)	5.81 (2.17)	1.18	0.24	0.25
Real	7.54 (1.73)	6.77 (1.80)	2.18	0.03*	0.44
Compelling	7.43 (1.77)	6.96 (1.80)	1.24	0.22	0.26
*Verbal thoughts in daily life*					
Frequency	5.22 (2.78)	4.81 (2.59)	0.61	0.54	0.15
Real	6.43 (2.75)	6.52 (1.71)	0.83	0.41	0.04
Compelling	6.40 (2.63)	6.71 (1.68)	0.18	0.86	0.13

SD, standard deviation. *P<0.50.

#### 3.3.1 Subjective domain of mental imagery

All results related to the subjective domain of mental imagery are detailed in Table 3. Participants with BD did not significantly differ from non‐clinical controls in their spontaneous use of imagery on the SUIS, and did not differ from non‐clinical controls in their rated frequency of thoughts in a verbal or visual modality over the past week.

Participants with BD did not significantly differ from non‐clinical controls in their imagery interpretation bias as assessed by pleasantness or vividness ratings on the AST‐D. Non‐clinical control participants reported a higher number of benign homographs which was marginally significant compared to those with BD. The two groups did not significantly differ in other interpretation bias ratings from the HIT.

On measures of emotional mental imagery, participants with BD scored higher on the PW self‐involvement scale compared to non‐clinical control participants. The two groups did not differ in any of the other PW task scales. Participants with BD reported a stronger impact of emotional prospective imagery on the IFES compared to non‐clinical controls. They also reported higher ratings of vividness and sense of experiencing for negative future images, and lower ratings of likelihood for positive future scenarios on the PIT. The two groups did not significantly differ on the remaining PIT scales.

All results on the MII are detailed in Table 3. For the time when their mood was most *low*, participants with BD rated their most significant mental image as more negative and more demotivating compared to non‐clinical controls. For the time when their mood was most *anxious*, participants with BD rated their most significant mental image as more negative, threatening and emotional compared to non‐clinical controls. They also rated overall thinking in mental images to be more frequent and more “real” compared to non‐clinical controls. For the time when their mood was most *high*, participants with BD rated their most significant image as more exciting compared to non‐clinical controls. They also rated overall mental imagery as more “real” compared to non‐clinical controls. Full results are reported in Table 4. Qualitative examples of significant mental images are reported in Table 5.

**Table 5:** Example of significant images for each affect state (anxiety and low and high mood) for participants with bipolar disorder and non‐clinical controls taken from the Mental Imagery Interview and mean emotional ratings of the significant images

	Bipolar disorder	Non‐clinical controls
Low mood	A suicide plan—extensive and intelligent. I would go to the college bar and take one of the CO_2_ bottles used to pump Guinness and take it back to my room. I would send an email to tell people not to come in and release the CO_2_ (pp 157)	Seeing the email rejecting you from the job (pp 131)
	Thinking about mold growing in the kitchen. The corners of the surfaces having mold, greeny gray mold. General disorder—lots of dirty crockery, lots of food. General horror. Smell of mold (pp 178)	What my mother looked like when healthy and well. What she looked like after a series of strokes (pp 269)
	Picture of a human brain with nasty pathology—fear about my own brain. Up close, almost immersive, not a scan section, being in the middle of it. Cavities with fluid in them, well lit, soft white yellow light. Quiet (pp 185)	Envisage driving into the carpark, walking upstairs and into the office and not feeling happy (pp 294)
Ratings: Mean (SD)	Demotivating**, c: 7.04 (2.23); Emotional^a^: 7.13 (2.27); Negative*, b: 2.13 (1.44)	Demotivating: 4.82 (2.59); Emotional: 6.77 (1.57); Negative: 2.96 (1.82).
Anxious state	Paranoid fear of future—teacher reprimanding me for not working. Expectation or need for punishment. Me alone in a classroom, teacher shouting, aggressive gestures, fingers pointing. Me sitting down then standing up. Height difference, I am being looked down upon physically and metaphorically (pp 153)	Best friend and me having a cup of coffee having an argument (pp 129)
	Husband's taken the children away. Seeing children with my husband, told to pack their bags and get in the car. They're confused, they're packing, doing what dad says. He's packing as well. I can see myself upset in the image (p 177)	Visiting a client during my shadowing day (pp 149)
	A man was cutting down a bush and I could see his gardening tool slipping and he cuts his arm off. Very vivid, it seems like my imagination running over, seems quite real. A lot of blood (pp 190)	Seeing boss call me to say I was being made redundant (pp 251)
Ratings: Mean (SD)	Threatening**, c: 7.00 (2.21); Emotional: **, c 7.69 (1.45); Negative**, c: 1.81 (1.04)	Threatening: 5.52 (2.25); Emotional: 6.24 (1.97); 2.86 (1.31)
High mood	Me in a very successful situation, written a brilliant book, receiving accolades. Critical acclaim in a paper. Image of me receiving award “He's so insightful” receiving it in front of friends and family (pp 155)	Trees, breeze, peace of the countryside (pp 124)
	Superb sex with someone utterly untouchable. See understanding and conversation and absolute everything being tuned in with each other. Huge praise coming your way, acceptance and appreciation. Somebody being as infatuated with you as you are them. Seeing a home and a setting where it is all going to happen, stuff gathered for you because everything is going to be possible (pp 163)	Image of standing in a doorway and chatting to everyone and they are all smiling back (friends) (pp 142)
	I can see images of things in general relativity, e.g. curved space time. Very real, I can build on that. Images are part of my work, problem solving for me. Rubber ball in a sheet, taking a 2D object and making it into 3D. I can see how the other dimensions work. You can write the algebra, visual equivalent of algebraic formula (pp 185)	Image of myself as a wise, guru‐like figure (pp 279)
Ratings: Mean (SD)	Exciting**, c: 8.06 (1.24); Emotional^a^: 7.16 (2.04); Positive^a^: 7.76 (1.80)	Exciting: 7.04 (1.54); Emotional: 6.92 (1.44); Positive: 7.80 (0.91)

pp, participant; SD, standard deviation.

*P<0.05; **P<0.01.

^a^0<*d*≤ 0.3.

^b^0.3<*d*≤0.6.

^c^ *d*>0.6.

#### 3.3.2 Cognitive (non‐emotional) stages of mental imagery

All results related to the cognitive (non‐emotional) stages of mental imagery are detailed in Table 2. Participants with BD did not significantly differ in their performance on any part of the imagery generation task, indicating no imagery abnormalities in the BD group in terms of imagery generation in a non‐emotional cognitive task.

Of the two imagery maintenance tasks, participants with BD had a higher recall rate on the visual STM task compared to those in the non‐clinical control group, indicating that participants with BD in this study had a greater likelihood of remembering visual target cues in a non‐emotional visual short‐term memory task. The groups did not differ significantly in memory precision. Instead, on the VPT task participants with BD did not significantly differ in accuracy scores from non‐clinical controls, suggesting that participants with BD in this study had no abnormalities in visual short‐term memory as assessed with the VPT.

Participants with BD did not significantly differ from non‐clinical controls in their accuracy or completion time on the LCC, indicating that participants with BD in this study had no dysfunctions in imagery inspection.

On imagery manipulation tasks, participants with BD did not significantly differ from non‐clinical controls on any indices of mental rotation speed, but on average had a higher error percentage compared to those in the non‐clinical control group. As, compared to non‐clinical controls, the BD group performed worse on the verbal fluency test, a task of executive function considered to be an indirect measure of general cognitive functioning, we tested if this could account for the higher error percentage on the MRT. The verbal fluency test total score was entered as a covariate in an ANCOVA testing group differences between participants with BD and non‐clinical controls in the error percentage of the MRT. After adding verbal fluency as a covariate, the main effect of group became marginally non‐significant [*F*(1, 75)=3.70, *P*=.058], indicating that the higher error rate of patients with BD on the MRT might be in part explained by deficits in general cognitive functioning.

Participants with BD did not differ from non‐clinical controls in their performance on the CMS. Over the two trials of the CMS, there were no group differences in the number trials judged as: recognizable (*P*=.49), creative (*P*=.08), having a poor correspondence (*P*=.34), or having no pattern (*P*=1.00). None of the participants used wrong parts in the pattern drawing.

### 3.4 Are mental imagery abnormalities specific to patients with BD?

Next we tested the specificity of findings to BD compared to individuals with unipolar depression and individuals with anxiety disorders (see the section ‘Statistical analysis’).

#### 3.4.1 Subjective domains of mental imagery

(ii‐a) Comparing BD depressed to unipolar depressed participants, differences were only detected on two items of the MII. For times when their mood was lowest, participants in the BD depressed group rated their most significant image as less demotivating (M=6.76, SD=2.30) than the unipolar depressed group (M=7.67, SD=2.20; *Z*=2.00, *P*=.05, *d*=0.40). Furthermore, for times when their mood was highest, participants in the BD depressed group rated their most significant image as more exciting (M=8.25, SD=1.22) than participants with unipolar depression (M=7.26, SD=1.61; *Z*=2.42, *P*=.02, *d*=0.69).

(ii‐b) There were no differences in emotional mental imagery (PW self‐involvement, *P*=.55; IFES total score, *P*=.33; PIT negative vividness, *P*=.42; PIT negative experiencing, *P*=.10; PIT positive likelihood, *P*=.32) comparing BD depressed participants with concurrent anxiety symptoms to participants with anxiety disorders.

#### 3.4.2 Cognitive (non‐emotional) stages of mental imagery

There were no differences in imagery manipulation (based on performance on the MRT) or in visual short‐term memory (based on recall rate scores on the STM task) between (ii‐a) BD depressed and unipolar depressed patients (MRT percentage errors, *P*=.82; STM recall rate, *P*=.25) or between (ii‐b) BD depressed patients with concurrent anxiety symptoms and anxious patients (MRT percentage errors, *P*=.70; STM recall rate, *P*=0.72).

### 3.5 Relation between mental imagery measures and depression, anxiety, BD phenotype, affective lability and general functioning

Given the lack of specificity of mental imagery abnormalities present in participants with BD, we next explored whether these imagery abnormalities are related to current affect states (QIDS, HAM‐D and BAI) and traits (MDQ and ALS) rather than diagnostic categories, and to levels of general functioning (FAST). Correlations between the clinical variables and measures of mental imagery are shown in *Supplementary Table S1*. To test the unique contribution of the associations between mental imagery variables and the clinical variables, we conducted a number of multiple regression analyses (see ‘Statistical analysis’), in which each imagery variable was predicted by the clinical variables (*Supplementary Table S2*). Only those imagery variables that showed significant group differences in the comparison between participants with BD and non‐clinical control participants (reported in Table 2) were included in these analyses.

#### 3.5.1 Cognitive (non‐emotional) stages of mental imagery

##### BD phenotype

Across all groups, higher levels of hypomanic experiences (measured by the MDQ scores) were associated with worse performance on *imagery manipulation* as measured by a higher error rate on the MRT task [β=0.249, *t*(122)=2.835, *P*=.005].

##### Affective lability

Across all groups, higher levels of affective lability (measured by scores on the ALS) were associated with better performance on *imagery maintenance* as measured by a higher recall rate on the STM task [β=0.229, *t*(114)=2.513, *P*=0.013].

#### 3.5.2 Subjective domains of mental imagery

##### BD phenotype

Across all groups, higher levels of hypomanic experiences (measured by the MDQ scores) were associated with higher ratings on how threatening the most significant image was at times of anxious affect on the MII [β=0.190, *t*(114)=2.068, *P*=.041], and on how exciting the most significant image was at times of elated mood [β=0.221, *t*(119)=2.467, *P*=.015].

##### Affective lability

Across all groups, higher affective lability scores (measured on the ALS) were associated with greater impact of emotional prospective imagery scores on the IFES [β=0.293, *t*(119)=3.43, *P*=.001]. Higher affective lability levels were also associated with higher ratings on the MII of how negative the most significant image was at times of low mood [β=−0.333, *t*(112)=3.740, *P*<.001], and how frequently participants were thinking in mental images at times of anxious affect [β=0.250, *t*(125)=2.885, *P*=.005] On the PW task, higher affective lability was associated with higher ratings of how self‐involved participants felt when generating picture−word combinations [β=0.189, *t*(122)=2.466, *P*=.015].

##### Anxiety

Across all groups, higher anxiety scores (measured on the BAI) were associated with greater impact of emotional prospective imagery scores on the IFES [β=0.415, *t*(119)=4.23, *P*<.001]; higher vividness ratings of negative events on the PIT [β=0.343, *t*(124)=3.54, *P*=.001], and a stronger sense of experiencing imagined negative events on the PIT [β=0.293, *t*(124)=2.530, *P*=.013].

##### Low mood

Across all groups, lower depression scores (measured on the QIDS) were associated with higher likelihood ratings of positive imagined events to happen on the PIT [β=−0.431, *t*(125) =5.344, *P*<.001]. Higher depression scores instead were significantly associated with ratings of how demotivating the most significant image was at times of low mood [β=0.305, *t*(111)=3.371, *P*=.001].

##### General functioning

Across all groups, lower general functioning (measured using the FAST scores) was associated with greater impact of emotional prospective imagery scores on the IFES [β=0.259, *t*(19)=2.64, *P*=.009]; and with how real mental images were rated at times of anxious affect on the MII [β=0.244, *t*(123)=2.792, *P*=.006].

Clinical and functioning variables across all groups were not related to how real mental images were rated at times of high mood [*F*(6,118)=0.96, *P*=.46, *R* ^2^=.05].

## 4 Discussion

### 4.1 Summary of main findings

Our study investigated, first, whether BD is associated with abnormalities in mental imagery compared to non‐clinical controls; second, whether any such abnormalities are specific to BD when compared to clinical control groups matched for depression and anxiety. Further, we explored whether abnormalities in mental imagery are associated with clinical variables *across* diagnostic groups. Results show that, compared to non‐clinical controls, individuals with BD show largely intact performance on experimental tasks measuring the cognitive (non‐emotional) stages of mental imagery. However, in the subjective domains of mental imagery, compared to non‐clinical controls, individuals with BD do show some abnormalities in emotional mental imagery: namely, a greater impact of intrusive prospective imagery in daily life, more vivid and “real” negative images on a prospective imagery task, and higher levels of self‐involvement in imagery on a picture−word task. Moreover, on a phenomenological interview about times of intense affect (anxious, depressed, or high), it was characteristics of mental imagery, but not of verbal thoughts, that distinguished individuals with BD from non‐clinical controls. Results further indicate that, when compared to clinical control groups matched for depression and anxiety, abnormalities in emotional mental imagery were not specific to BD but associated with increasing psychopathology.

Interestingly, our results show that, across all clinical and non‐clinical groups, mental imagery abnormalities are associated with severity of depression and anxiety, as well as with BD phenotype and affective lability traits, and general functioning. This raises the possibility that the subjective experience of highly emotional mental imagery (assessed by a range of measures across different affect states) is a *transdiagnostic* feature of psychopathology, and associated in particular with affective lability across clinical and non‐clinical populations. This finding is particularly interesting given that affect lability (including concurrent anxiety) represents a particularly challenging feature *across* different mental disorders.

### 4.2 Emotional mental imagery in BD

We replicated previous findings that individuals with BD (euthymic and depressed combined) experience a greater impact of intrusive prospective mental imagery in everyday life,^7^ and perceive prospective negative images as more vivid in an experimental task compared to non‐clinical controls.^7^ Further, we extended these findings in that our sample of participants with BD also reported more real (greater “sense of experiencing”) prospective negative mental images and perceived imagined positive events as less likely to occur compared to non‐clinical controls. Consistent with a greater sense of experiencing anxiety for negative prospective images, compared to non‐clinical controls, participants with BD also felt more self‐involved when spontaneously generating mental images by combining negative pictures and words.

Unlike previous studies, we did not find evidence of greater spontaneous use of non‐emotional mental imagery in BD compared to non‐clinical controls, although mean values were in the same direction as in a previous study.^7^ This suggests that differences in spontaneous tendency to visualize are likely to be small and test of replication in larger samples is needed to verify these inconsistencies.

Overall our BD sample reported imagery abnormalities particularly for prospective imagery and during anxious affect. This is consistent with the relationship between anxiety and future thinking.^46^ As prospection plays an important role in regulating emotions and behavior,^47^ it is possible that these abnormalities in prospective imagery (although not limited to BD; see below) contribute to emotional and behavioral dysregulation typical of BD.^6^ It could be fruitful to investigate the effect of prospective imagery on the presence and severity of comorbid anxiety, which is a key clinical feature in BD.^48^, ^49^ For example, one participant with BD reported that when most anxious they repeatedly experience vivid negative images of embarrassing themselves at a social event; the images feel so real that they further fuel anticipatory anxiety to the point of making them avoid attending the event. A better understanding of prospective anxiety‐inducing imagery in BD may also have implications for therapy, given the challenge of treating anxiety in this disorder.^18^, ^48^ Future studies could investigate whether the experience of emotional mental imagery in BD differs depending on the type of anxiety comorbidity, following current cognitive accounts of anxiety disorders where imagery is predominant, such as social anxiety,^16^ or irrelevant if not suppressed, such as general anxiety disorder.^50^

### 4.3 Cognitive stages of mental imagery in BD

Finally, and novel to the literature (as called for by Pearson et al.^11^), the absence of major dysfunctions in the cognitive (non‐emotional) stages of mental imagery suggests that there are no deficits in the ability to generate, manipulate, and recall images. Interestingly, our BD sample also showed a greater likelihood of recalling the target cues in one of the visual short‐term memory tasks compared to non‐clinical controls. Thus, individuals with BD appear to have an overall intact functioning or even an “advantage” in this aspect of imagery processing. Therefore, drawing on mental imagery techniques^18^, ^51^ could be a successful strategy in treatment interventions for BD where other cognitive processes may be impaired (as in our sample with reduced verbal fluency/executive function performance).^52^

### 4.4 Mental imagery abnormalities as a transdiagnostic phenomenon

Unlike previous studies comparing BD and unipolar depressed patients,^8^, ^10^ no differences emerged between our clinical groups in prospective imagery measures. In fact, across the whole sample combined, prospective imagery abnormalities (on IFES total and PIT negative scenarios scores) were associated with severity of anxious symptomatology and affective lability traits. This suggests that inconsistencies between studies of clinical groups may be accounted for by the relative distribution of affective lability traits and concurrent anxiety in the samples.

Consistent with previous data,^10^ depressed participants with BD rated their most significant image at times of high mood as more exciting compared to participants with unipolar depression. This may reflect both an association between mania and positive mental imagery (even at times of depressed mood) and a deficit in positive mental imagery in unipolar depression^51^, ^53^, ^54^. The finding is also consistent with recent neuroimaging evidence showing that participants with BD and unipolar depression present different neural responses to positive stimuli only while depressed.^55^ We did not replicate previous evidence of negative images being more compelling in BD compared to unipolar patients.^8^ This discrepancy might be accounted for by less severely depressed samples in the present study or may suggest that greater compellingness might be specific to suicidal flashforwards in patients with BD^8^ rather than any image during low mood.

With regard to the cognitive (non‐emotional) stages of mental imagery, previous studies have reported biases in imagery generation and manipulation in unipolar depressed individuals compared to controls^13^, ^44^; however, these depression‐related abnormalities were only present in measures that index the sensory/response component of imagery tasks^13^ rather than specific imagery (e.g. spatial ability) processing biases. Therefore, discrepancies between studies may be explained by differences in sensory‐motor retardation symptoms between the samples.

Overall, our study indicates that mental imagery characteristics representing features of greater emotionality and intensity (e.g., greater intrusive imagery impact, vividness of negative images, and sense of realness of images) may represent a marker for general emotional psychopathology, and general functioning. This supports our idea that “bringing back the mind's eye” to psychiatric assessments^17^ could help identify clinical severity. Most importantly it can help clinicians to understand and normalize aspects of patients’ subjective experiences that may otherwise feel particularly alien and distressing (such as intrusive highly emotional mental images). Asking about mental images offers an alternative access to capturing distress in those patients who may struggle to communicate their subjective experiences via traditional verbal thoughts. The transdiagnostic relevance of mental imagery also highlights potential avenues for new treatment interventions: e.g. if depression scores relate to how likely positive future images feel, reverting positive imagery biases may be a useful target to improve mood.^36^, ^51^, ^56^

Our results on the association between BD phenotype and affective lability traits, and greater imagery frequency and emotionality, are in keeping with previous findings that individuals with a BD phenotype are more susceptible to intrusive imagery and to spontaneous use of imagery.^57^, ^58^ Interestingly, better performance in imagery maintenance via visual short‐term memory was also associated with affective lability. Future studies should investigate the relationship between biases in emotional mental imagery, visual short‐term memory function and emotional instability across psychopathology, including in other conditions where this is relevant such as borderline personality disorder. Overall, mental imagery biases could be conceptualized as a cognitive psychopathological dimension in line with most recent neuroscience dimensional approaches to understanding mental disorders (research domain criteria^59^). Future research should investigate how currently established cognitive and neural markers of emotional dysregulation and affective lability^60^, ^61^, ^62^ relate to abnormalities in emotional mental imagery described in our sample. Moreover, as affective lability often represents a therapeutic challenge, treatment innovation should explore the potential for using imagery‐focused interventions for emotional instability.^51^

## 5 Limitations and Conclusions

A limitation of our study is the absence of statistical correction for multiple comparisons. Moreover, we did not include a (hypo)manic BD group that would allow us to establish the presence of mental imagery abnormalities associated with mania state diagnosis. With regard to results from the MII, it should also be noted that these were based on retrospective accounts of times of intense affect and could have been subject to recall/memory biases. Future qualitative studies are needed to analyze in detail potential differences in the image contents exemplified in Table 5. Our data suggest that mental imagery abnormalities are typical of acute clinical states of anxiety and depression, but are also associated with traits of BD phenotype and affective lability. Future studies should include individuals recovered from unipolar depression and anxiety disorders to clarify if emotional mental imagery abnormalities also persist beyond acute depression/anxiety across psychopathology, as they do in our BD sample (euthymic and depressed). Moreover, as our clinical groups all presented moderate to high levels of both anxiety and depression, future studies could attempt to tease apart the association between mental imagery abnormalities and anxiety/depression, although this may be a challenge given the high co‐occurrence of these symptoms in emotional disorders. Nevertheless, the regression analyses across all groups in our sample suggest a greater impact of anxiety on mental imagery characteristics. Finally, longitudinal rather than cross‐sectional studies should further investigate stability and change of mental imagery abnormalities in BD over the course of illness. Future studies could also compare individuals with bipolar I and II disorder using sufficiently powered samples.

In conclusion, this first comprehensive investigation of a range of mental imagery measures in BD compared to both non‐clinical and clinical controls confirms that imagery abnormalities are present in patients with BD in the emotional aspects of mental imagery, while the cognitive processes underpinning mental imagery experience remain largely intact. Biases in emotional mental imagery appear as a transdiagnostic feature of our clinical groups matched on depression and anxiety levels related to clinical dysfunction. We suggest that imagery abnormalities are a transdiagnostic processes driving affective lability, and that imagery can be targeted via novel psychological treatment techniques. Imagery‐focused techniques hold promise across psychiatric disorders,^17^ including adding treatment value to BD.^18^

## Disclosures

The authors of this paper do not have any commercial associations that might pose a conflict of interest in connection with this manuscript.
