# Supplementary material for: An investigation of mental imagery in bipolar disorder: Exploring “the mind's eye”
Source: Bipolar Disord. 2016 Dec 20;18(8):669–83. doi: 10.1111/bdi.12453 (PMC5299482; doi:10.1111/bdi.12453)
Supplement: Supplementary file 1 [file BDI-18-669-s001.doc]

**Supplementary material**

**Supplementary Table 1 – Correlation between clinical variables and imagery measures for the whole sample combined**

|  | **QIDS** | **HAM-D** | **BAI** | **MDQ** | **ALS** | **FAST** |
| --- | --- | --- | --- | --- | --- | --- |
| **Imagery Maintenance** |  |  |  |  |  |  |
| STM recall | 0.11 | 0.13 | 0.09 | 0.16 | .24^**^ | 0.09 |
| **Imagery Manipulation** |  |  |  |  |  |  |
| MRT Percentage Errors | 0.17 | .18^*^ | .19^*^ | .25^**^ | .18^*^ | .18^*^ |
| **Emotional Mental Imagery** |  |  |  |  |  |  |
| IFES Total Score | .64** | .62** | .73** | .21* | .59** | .67** |
| PIT Negative Vividness | .35** | .37** | .42** | 0.13 | .36** | .36** |
| PIT Negative Experiencing | .38** | .35** | .43** | 0.12 | .35** | .41** |
| PIT Positive Likelihood | -.42** | -.42** | -.27** | -0.12 | -.22* | -.40** |
| MII low mood significant image:  Demotivating | .34^**^ | .29^**^ | .34^**^ | .25^**^ | .32^**^ | .24^*^ |
| Negative | -.25^**^ | -.19^*^ | -.26^**^ | -.22^*^ | -.34^**^ | -0.15 |
| MII anxious mood significant image:  Threatening | 0.00 | -0.03 | -0.03 | .18^*^ | 0.14 | 0.02 |
| Emotional | 0.08 | 0.09 | 0.03 | 0.14 | 0.13 | 0.11 |
| Negative | -0.03 | 0.05 | 0.07 | -0.14 | -0.10 | 0.04 |
| MII anxious affect - realness of images | .19^*^ | .18^*^ | .24^**^ | 0.13 | .23^**^ | .25^**^ |
| MII anxious mood - time thinking in images | 0.16 | .19^*^ | 0.15 | 0.17 | .26^**^ | 0.17 |
| MII high mood significant image:  Exciting | 0.01 | -0.01 | 0.02 | .24^**^ | 0.17 | 0.06 |
| MII high mood - realness of images | 0.03 | 0.06 | 0.08 | 0.17 | 0.05 | 0.13 |
| PWT self-involvement | 0.28 | 0.17 | 0.29 | 0.21 | 0.34 | .31** |

*Note.* ** = p<.01; * = p<.05 (2-tailed); STM = *Short-Term Memory task (35)*; MRT = *Mental Rotation Task (39)*; IFES *= Impact of Future Events Scale (16)*; PIT *= Prospective Imagery Task*; MII = *Mental Imagery Interview (8)*; PW = *Picture Word task (43).*

**Supplementary Table 2 – Regression models: mood, anxiety, bipolar phenotype, affective lability and general functioning as predictors of imagery biases across clinical and non-clinical groups**

|  | **Model 1 (standardized betas)** | | | | | | **Final model (standardized betas)** | | | | | |
| --- | --- | --- | --- | --- | --- | --- | --- | --- | --- | --- | --- | --- |
|  | **QIDS** | **HAMD** | **BAI** | **MDQ** | **ALS** | **FAST** | **QIDS** | **HAMD** | **BAI** | **MDQ** | **ALS** | **FAST** |
| **Imagery Maintenance** |  |  |  |  |  |  |  |  |  |  |  |  |
| STM recall | -0.10 | 0.20 | -0.12 | 0.02 | 0.27 | -0.03 | - | - | - | - | .23^*^ | - |
| **Imagery Manipulation** |  |  |  |  |  |  |  |  |  |  |  |  |
| MRT Percentage Errors | 0.00 | 0.05 | 0.13 | .25* | -0.06 | 0.01 | - | - | - | .25^**^ | - | - |
| **Emotional Mental Imagery** |  |  |  |  |  |  |  |  |  |  |  |  |
| IFES Total Score | -0.11 | 0.09 | .42** | -0.09 | .29* | .26* | - | - | .44** | - | .22** | .24** |
| PIT Negative Vividness | -0.17 | 0.18 | 0.27 | -0.04 | 0.20 | 0.08 | - | - | .34** | - | 0.16 | - |
| PIT Negative Experiencing | -0.03 | -0.01 | 0.25 | -0.04 | 0.14 | 0.21 | - | - | .29* | - | - | 0.21 |
| PIT Positive Likelihood | -0.29 | -0.20 | 0.24 | -0.04 | 0.02 | -0.21 | -.43** | - | - | - | - | - |
| MII low mood significant image:  Demotivating | 0.35 | -0.02 | 0.21 | 0.16 | 0.06 | -0.27 | .30^**^ | - | - | 0.16 | - | - |
| Negative | -0.30 | 0.06 | -0.15 | -0.09 | -0.22 | 0.31 | - | - | - | - | -.33^**^ | - |
| MII anxious mood significant image:  Threatening | 0.01 | -0.08 | -0.08 | -0.14 | 0.12 | 0.03 | - | - | - | .19^*^ | - | - |
| Emotional | -0.04 | 0.10 | -0.15 | 0.07 | 0.10 | 0.09 | - | - | - | - | - | - |
| Negative | -0.43 | -0.23 | 0.12 | -0.16 | -0.03 | 0.19 | - | - | - | -0.16 | - | - |
| MII anxious affect - realness of images | 0.13 | -0.02 | 0.14 | 0.00 | 0.14 | 0.19 | - | - | - | - | - | .24^**^ |
| MII anxious mood - time thinking in images | -0.15 | 0.23 | -0.11 | 0.00 | .26* | 0.05 | - | - | - | - | .25^**^ | - |
| MII high mood significant image:  Exciting | -0.08 | -0.13 | -0.03 | 0.17 | 0.12 | 0.15 | - | - | - | .22^*^ | - | - |
| MII high mood - realness of images | -0.14 | -0.03 | 0.07 | 0.17 | -0.12 | 0.23 | - | - | - | - | - | - |
| PW Self-involvement | 0.22 | -.39* | 0.11 | 0.03 | 0.19 | 0.23 | - | - | - | - | .24* | 0.19 |

*Note.* Multiple regression analyses were conducted with each of the mental imagery measures as dependent variable and with all clinical variables entered as predictor variables simultaneously. Only those mental imagery characteristics where a difference between the BD group and non-clinical controls had been detected (see Tables 2, 3a, 3b) were analysed. Non-significant predictors were then removed from the model stepwise until only significant predictors remained.

** = p<.01; * = p<.05 (2-tailed); STM = *Short-Term Memory task (35)*; MRT = *Mental Rotation Task (39)*; IFES *= Impact of Future Events Scale (16)*; PIT *= Prospective Imagery Task (50)*; MII = *Mental Imagery Interview (8)*; PW = *Picture Word task (43).*
